# Effects of moxibustion therapies on immune function in cancer animal models: a systematic review and meta-analysis

**DOI:** 10.3389/fimmu.2026.1724707

**Published:** 2026-03-12

**Authors:** Xinyue Liang, Yu Ma, Xiaoqi Zhang, Mai Zhang, Lijia Pan

**Affiliations:** College of Acupuncture-Moxibustion and Tuina, Hebei University of Chinese Medicine, Shijiazhuang, Hebei, China

**Keywords:** animal models, cancer, immunity, meta-analysis, moxibustion

## Abstract

**Background:**

Moxibustion is a key component of traditional Chinese medicine and is widely employed in clinical practice as an adjunctive therapy for cancer. Preclinical studies have demonstrated that moxibustion can modulate host immune function, improve the tumor immune microenvironment, and suppress tumor growth. This study aims to evaluate the effects of moxibustion on immune function in animal models of cancer.

**Methods:**

A systematic literature search was conducted to identify all animal studies on moxibustion therapy for cancer in English-language databases (PubMed, Cochrane Library, Embase, and Web of Science) and Chinese-language databases (CNKI, Wanfang, CBM, and VIP). Data extraction was performed independently by two reviewers. Statistical analyses were carried out using RevMan 5.4 software. Potential publication bias was assessed using Egger’s test and funnel plots.

**Results:**

The initial literature search identified a total of 2639 potentially relevant studies, of which 48 met the predefined inclusion criteria. Meta-analysis revealed that moxibustion therapy significantly inhibited tumor growth in animal models. Compared with the control group, moxibustion was associated with a significant reduction in tumor volume (SMD = -1.79; 95% CI [-2.59, -0.99]; *P* < 0.0001) and tumor weight (SMD = -1.48; 95% CI [-1.88, -1.08]; *P* < 0.00001). The spleen index was elevated (SMD = 0.69; 95% CI [0.08, 1.29]; *P* = 0.03), IL-2 levels were increased (SMD = 1.71; 95% CI [0.88, 2.55]; *P* < 0.0001), IFN-γ levels were elevated (SMD = 1.14; 95% CI [0.72, 1.56]; *P* < 0.00001), while IL-6 levels showed a decreasing trend (SMD = -0.73; 95% CI [-2.42, 0.96]; *P* = 0.40), and TNF-α levels were elevated (SMD = 0.24; 95% CI [-0.81, 1.28]; *P* = 0.66).

**Conclusion:**

Although this study has certain limitations, the findings indicate that moxibustion therapy exerts an inhibitory effect on tumor growth in animal models of nine cancer types, including hepatocellular carcinoma, lung cancer, gastric cancer, sarcoma, breast cancer, colon cancer, rectal cancer, lymphoma, and colorectal cancer. Moxibustion also increases the spleen index, elevates levels of anti-tumor immune cytokines such as IL-2, IFN-γ, and TNF-α, suppresses the pro-inflammatory cytokine IL-6, and enhances host immune function.

**Systematic review registration:**

https://www.crd.york.ac.uk/prospero/, identifier CRD42024564223.

## 1 Introduction

According to the data provided by the International Agency for Research on Cancer (IARC), in 2022, there were approximately 19.96 million new cancer cases worldwide, resulting in about 9.7 million deaths. Lung cancer was the leading cause of cancer-related deaths (accounting for 18.7% of all cancer deaths), followed by colorectal cancer (9.3%), liver cancer (7.8%), and female breast cancer (6.9%). The global incidence and mortality rates of cancer are increasing rapidly. It is projected that by 2050, the number of new cancer cases will reach 35 million, an increase of 77% compared to the 2022 level (1). Although traditional treatment methods such as surgery, radiotherapy and chemotherapy play a significant role in cancer treatment, they have many limitations, including significant side effects, easy development of drug resistance, and decline in patients’ quality of life (2–5).

The occurrence and progression of tumors are closely associated with the host immune system. Traditional Chinese Medicine (TCM) exerts anti-tumor effects through modulation of immune function and has demonstrated unique advantages in cancer therapy. Accumulating evidence indicates that TCM interventions can enhance cancer immunotherapy by downregulating the expression of PD-1 and PD-L1, modulating T cell function—including promotion of T cell proliferation, enhancement of effector T cell cytotoxicity, increased CD4^+^ T and CD8^+^ T cell ratios, and reduction in regulatory T cell (Treg) populations—improving the tumor immune microenvironment by elevating levels of IFN-γ and TNF-α while reducing pro-inflammatory and immunosuppressive cytokines such as IL-1β, IL-6, and TGF-β—and regulating gut microbiota composition (6). Clinical studies have demonstrated that acupuncture and moxibustion can promote a typical Th1 cell shift, increase the plasma level of IFN-γ, and reduce the levels of IL-4 and IL-6, thereby enhancing the body’s capacity to generate effective anti-tumor immune responses (7). Moxibustion, as a fundamental component of TCM therapy, exerts therapeutic effects by promoting the circulation of qi and blood through warming the meridians, regulating physiological balance, and enhancing the body’s defensive function while dispelling pathogenic factors. It has been widely utilized in clinical practice as an adjunctive therapy for cancer (8), demonstrating efficacy in mitigating adverse effects associated with radiotherapy and chemotherapy (9, 10), reducing recurrence and metastasis rates (11), and improving the quality of life among cancer patients (12).

Tregs play a critical role in suppressing the proliferation of effector T cells, thereby facilitating tumor immune escape (13). Preclinical animal studies have demonstrated that moxibustion can effectively restore immune homeostasis within the tumor microenvironment by downregulating Tregs and associated immunosuppressive factors (IL-10, TGF-β1), while simultaneously upregulating Th17 cells and their related effector molecules (IL-17A) with anti-tumor activity, leading to inhibition of tumor growth (14). Compared to cisplatin monotherapy, the combination of moxibustion and cisplatin not only exerts stronger anti-tumor effects but also enhances anti-tumor immune responses through multiple mechanisms. This combinatorial approach significantly promotes Th1 cell infiltration into tumor tissues and further recruits CD8^+^ T cells, CD4^+^ T cells, and Th9 cells via the secretion of IL-9 and IL-21, thereby potentiating CD8^+^ T cell-mediated specific anti-tumor immunity. Furthermore, the combination therapy may induce polarization of M1-type macrophages; these activated macrophages enhance tumor infiltration of CD8^+^ T cells, Th1, and Th9 cells by secreting pro-inflammatory cytokines and remodeling the immune microenvironment, ultimately generating a robust anti-tumor immune response through synergistic actions of multiple cytokines (15).

Although moxibustion has shown promise in inhibiting tumor growth, enhancing immune function, and improving the efficacy of chemotherapy in animal models (8, 16), most existing studies are fragmented and lack systematic investigation into underlying mechanisms or robust experimental evidence. To date, no comprehensive meta-analysis or systematic quality assessment of preclinical animal studies on the antitumor effects of moxibustion has been published (17). Therefore, this study conducted a systematic review and meta-analysis of moxibustion therapy in animal tumor models, with the aim of comprehensively summarizing existing preclinical evidence, evaluating the effects of moxibustion on immune function in tumor-bearing animals through meta-analysis, and identifying key methodological variables critical for ensuring reproducibility in future research.

## 2 Materials and methods

This study was conducted in accordance with the Preferred Reporting Items for Systematic Reviews and Meta-Analyses (PRISMA) guidelines (18). The procedures and study inclusion criteria were prospectively registered in PROSPERO (CRD42024564223).(https://www.crd.york.ac.uk/prospero/).

### 2.1 Literature search strategy

A systematic search was conducted to identify articles published in electronic databases, including PubMed, the Cochrane Library, Embase, Web of Science, China National Knowledge Infrastructure (CNKI), Wanfang Database, VIP Database, and the Chinese Biomedical Literature Database (CBM), up to October 23, 2024. The literature was screened using the following MeSH terms and keywords: “Moxibustion”, “Neoplasms” [MeSH], and “Animals” [MeSH]. These terms were combined in various configurations using the Boolean operators “AND”, “OR”, and “NOT”. Two researchers (LXY, MY) independently performed the eligibility assessment of the retrieved articles. Discrepancies were resolved through discussion to achieve consensus. Only published articles were included in this study.

### 2.2 Inclusion and exclusion criteria

The inclusion criteria were established according to the PICOS framework (Population, Intervention, Comparison, Outcome, and Study Design) and are specified as follows:

1. Animal models: studies using experimental animals with induced tumor models, including mice, rats, rabbits, and other rodents;
2. Intervention: moxibustion techniques, including mild-warm moxibustion, grain-sized moxibustion, moxa cone moxibustion, heat-sensitive moxibustion, ginger-separated moxibustion, and direct moxibustion, among others;
3. Outcomes: primary outcome measures included tumor volume, tumor weight, tumor inhibition rate, and spleen index; secondary outcome measures included tumor necrosis factor (TNF-α), interleukin-2 (IL-2), interferon-γ (IFN-γ), and interleukin-6 (IL-6);
4. Study design: no restrictions on tumor type, moxibustion dosage, or treatment duration.

Exclusion criteria are as follows:

Reviews, commentaries, conference abstracts, case reports, clinical studies, and *in vitro* experiments;
Studies that do not use tumor or cancer models, lack a control group, or apply moxibustion in combination with other interventions;
Duplicate data or duplicated publications;
Insufficient or unsuitable data were provided for inclusion in the literature analysis, or the analytical methods employed were methodologically flawed;
Non-English and non-Chinese language publications.

### 2.3 Data extraction

In this study, two authors (Liang Xinyue and Ma Yu) independently extracted data from each included study using a pre-defined standardized form. All discrepancies were resolved through discussion with the corresponding author. Data extraction covered the following domains: 1) basic study information, limited to the primary author and year of publication; 2) animal characteristics, including strain, sex, body weight, and age; 3) experimental design details, encompassing modeling methods, tumor cell lines and cancer types, moxibustion intervention protocols, treatment dosage, duration of therapy, and number of animals per group; 4) outcome measures, including tumor volume, tumor weight, tumor inhibition rate, spleen index, tumor necrosis factor-α (TNF-α), interleukin-2 (IL-2), interferon-γ (IFN-γ), and interleukin-6 (IL-6). For numerical data not directly available, Origin 2025 was used to extract values from relevant graphs via graphic digitization and convert them into analyzable formats.

### 2.4 Literature quality assessment

The SYRCLE tool for risk of bias assessment was employed to evaluate the methodological quality of the included studies at the individual study level, thereby enabling a systematic appraisal of their potential biases (19). Assessment results were categorized as “low risk” “high risk” or “unclear risk” reflecting the presence or absence of factors that may introduce bias: “low risk” indicates adequate implementation of key methodological safeguards and a low likelihood of bias; “high risk” indicates failure to implement critical protections, leading to a high likelihood of bias; “unclear risk” indicates insufficient reporting, preventing a clear judgment of bias risk. To evaluate potential publication bias and result robustness, funnel plot analysis, subgroup analysis, and sensitivity analysis were conducted. The assessment was performed independently by two authors (Liang Xinyue and Ma Yu), with disagreements resolved through discussion or consultation with a third reviewer.

### 2.5 Statistic analysis

Statistical analysis was performed using RevMan version 5.4. For continuous variables, due to the different measurement methods and units, the standardized mean difference (SMD) and its 95% confidence interval (CI) are used as the effect size indicators. The overall effect was assessed using the Z test, and statistical significance was defined as *P* < 0.05. Between-study heterogeneity was evaluated using the chi-squared test, with a significance level set at *P* < 0.10. Heterogeneity was assessed using the I² statistic: an I² value exceeding 50% was considered indicative of substantial heterogeneity, and a random-effects model was therefore applied; otherwise, a fixed-effects model was used. Sensitivity analysis was conducted by sequentially excluding each included study to systematically evaluate the robustness of the pooled results. Publication bias was visually inspected using funnel plots to detect potential asymmetry across outcome indicators. Furthermore, for meta-analyses including 10 or more independent studies, Egger’s regression test, as proposed by Sterne et al. (20), was performed to provide a quantitative assessment of publication bias.

## 3 Results

### 3.1 Literature search results

**Figure 1:**
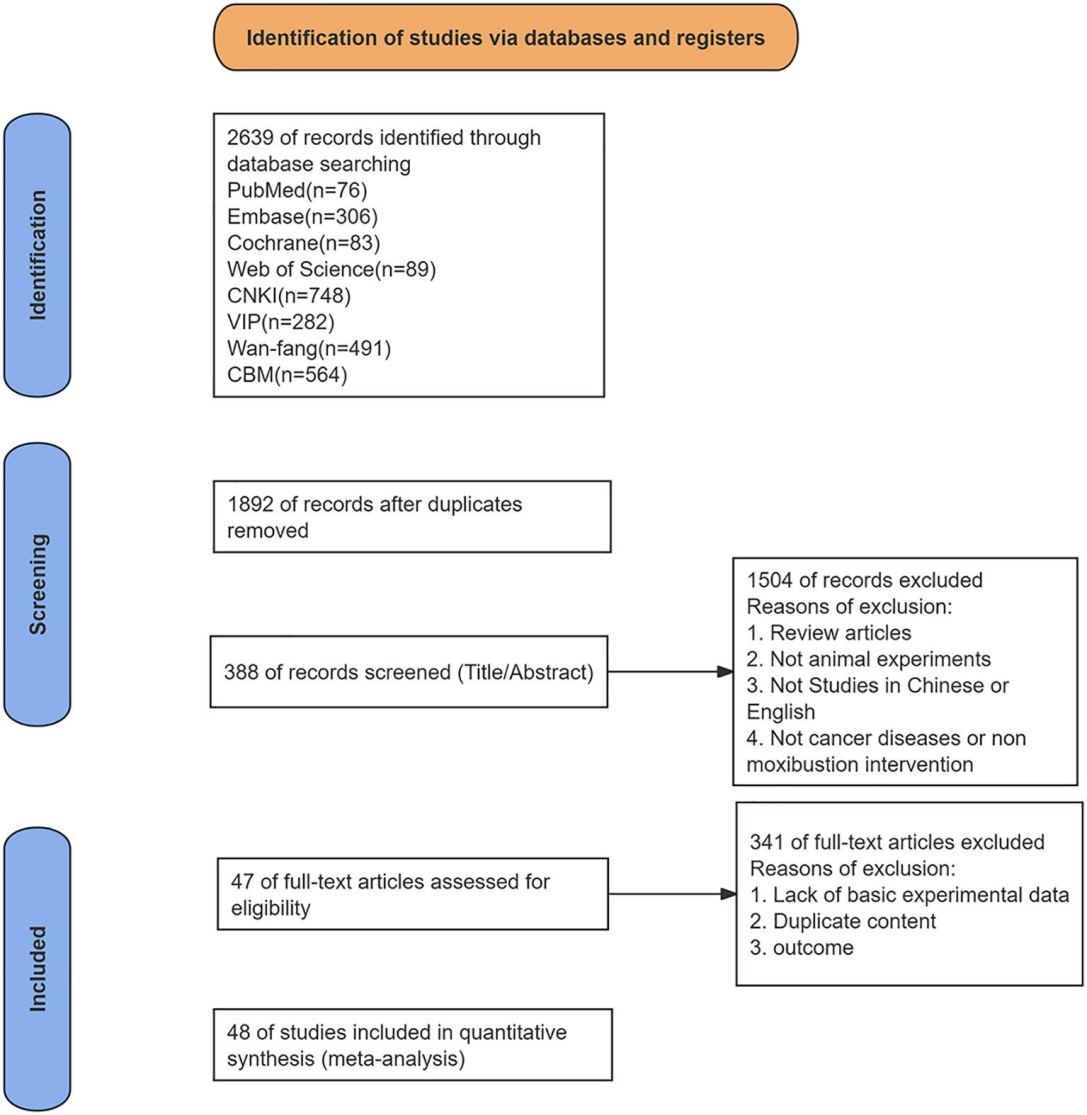
Flowchart of the PRISMA method.

A total of 2,639 potentially relevant records were identified through electronic database searches. After removing 747 duplicates, 1,845 studies were excluded during the initial screening phase for not meeting the predefined inclusion criteria. In cases where the same research content was published as both a journal article and a dissertation, data from the peer-reviewed journal publication were prioritized to avoid duplication of information. Ultimately, 47 publications encompassing 48 independent studies were included in the meta-analysis. The literature screening and selection process strictly adhered to the Preferred Reporting Items for Systematic Reviews and Meta-Analyses (PRISMA) guidelines and is summarized in the flow diagram presented in **Figure 1**.

### 3.2 Basic characteristics of the included studies

**Table 1** summarizes the detailed characteristics of the included studies. A total of 48 studies were included, involving seven animal strains: BALB/c mice (n = 10), C57BL/6 mice (n = 11), C57BL/6J mice (n = 6), SD rats (n = 6), Wistar rats (n = 3), Kunming mice (n = 10), and NIH mice (n = 2). The sample size per group typically ranged from 6 to 10 animals. Nine distinct animal tumor models were utilized, including gastric cancer (n = 6), lung cancer (n = 11), colon cancer (n = 1), rectal cancer (n = 1), colorectal cancer (n = 1), hepatocellular carcinoma (n = 12), sarcoma (n = 13), breast cancer (n = 1), and lymphoma (n = 2). The moxibustion interventions consisted of direct moxibustion (n = 23), indirect moxibustion (n = 2), suspended moxibustion (n = 17), and other methods (n = 6). In most studies, the intervention duration was at least two weeks, with treatments administered on a daily or alternate-day basis.

**Table 1:** Characteristic of studies included in systematic review and meta-analysis.

Number	Study(year)	Disease	Species and strains	Gender	Age	Weight	Cell line	Acupoint	Intervention	Frequency	Detection sites	Detection method	Outcome index
1	Xuejun Wang, 2024 (21)	RC	C57BL/6	M	6-8W	20 ± 2g	(Intraperitoneal injection of AOM)	ST25	HPM	3/w,60d	Intestinal epithelial cells	ELISA	TV,TNF-α
2	Yanting Cheng, 2023 (22)	HCC	C57BL/6	M	6-8W	16 ± 2g	Hepa1-6	DU14,ST36,SP6	CM	1/d,10d	Serum	ELISA	TW,TNF-α,IL-2,TIR
3	Ni Mao, 2023 (23)	LC	C57BL/6	F	6-8W	18-22g	Lewis	ST36,GV4	SM	4/w,2w	NR	NR	TV,TWTIR
4	Shanshan Lu, 2022 (24)	LC	C57BL/6	M	6-7W	20 ± 2g	Lewis	ST36	SM	5/w,2w	NR	NR	TV,TW
5	Tao Zhu, 2022 (25)	HCC	C57BL/6J	M	4–6 W	16–18 g	Hepa1-6	DU14,ST36,SP6	CM	1/d,10d	NR	NR	TV,TW,spleen index,TIR
6	Lijun Li-1, 2021 (26)	Sarcoma	C57BL/6J	M/F	5-7W	NR	S180	Tumor	SM	1/d,14d	Serum	Luminex liquid suspension chip	TW,IL-2,IFN-γ
7	Lijun Li-2, 2021 (27)	Sarcoma	C57BL/6J	M/F	5-7W	20 ± 3g	S180	Tumor	SM	1/d,14d	NR	NR	TW
8	Lijun Li-3, 2021 (28)	Sarcoma	C57BL/6	M/F	NR	18-20g	S180	Tumor	SM	1/d,14d	NR	NR	TW
9	Yujiao Jiang, 2020 (29)	LC	C57BL/6	M	8W	21-25g	Lewis	ST36,CV4	LM	1/d,15d	Serum	ELISA	TV,IFN-γ
10	Junlin Shi, 2020 (30)	GC	SD	M	NR	180-220g	Walker-256	CV12,CV4,ST36,BL20,BL21	SM	1/d,14d	NR	NR	TV,TW,TIR
11	Xiaobo Wu, 2020 (31)	LC	C57BL/6	M	8 W	18-20g	Lewis	ST36,CV4	LM	1/d,15d	Serum	ELISA	TV,TW,spleen index,IFN-γ
12	Ning Xue, 2020 (32)	BC	BALB/C	F	NR	18-22g	4T1	ST36	CM	1/d,2w	Serum	ELISA	TV,TW,spleen index,IL-2,IFN-γ,TIR
13	Bin Wang, 2020 (15)	LC	C57BL/6	M	6W	18-24g	Lewis	ST36	SM	5/w,2w	NR	NR	TV,TW
14	Huan Zhao, 2019 (33)	GC	SD	M	NR	180-220g	Walker-256	CV12,CV4,ST36,BL20,BL21	SM	1/d,2w	Skeletal muscle	ELISA	TNF-α,IL-6
15	Yupan Chen, 2019 (34)	GC	SD	M	NR	180-220g	Walker-256	CV12,CV4,ST36,BL20,BL21	SM	1/d,2w	NR	NR	TV,TW,TIR
16	Jing Tan-1, 2019 (35)	GC	SD	M	NR	200-240G	Walker-256	CV12,CV4,ST36,BL20,BL21	SM	1/d,21d	Tumor tissues	ELISA	TV,TNF-α,IFN-γ,TIR
17	Jing Tan-2, 2019 (36)	GC	SD	M	NR	220-240g	Walker-256	CV12,CV4,ST36,BL20,BL21	SM	1/d,21d	Tumor tissues	ELISA	TV,TNF-α,IL-6,IFN-γ,TIR
18	Ning Wang, 2018 (37)	HCC	Wistar	M	NR	200 ± 10g,	(Intraperitoneal injection of diethylnitrosamine)	BL18	CM	1/2d,10w	Serum	ELISA	TNF-α
19	Shibo Chen, 2018 (38)	LC	C57BL/6	M	4-5W	20 ± 2g	Lewis	LU1	CM	1/d,2w	NR	NR	TV,TW,TIR
20	Nan Wang, 2017 (39)	GC	SD	M/F	NR	NR	SGC7901	CV12,ST36	CM	1/d,15d	NR	NR	TW,TIR
21	Hong Liu, 2017 (40)	LC	BALB/C	M	4-6W	18-20g	Lewis	ST36	CM	1/d,10d	NR	NR	TV
22	Xue Zhang, 2016 (41)	LC	C57BL/6	M	4-6W	18-20g	Lewis	ST36	CM	1/d,10d	Serum	ELISA	TV,IL-6,
23	Hongda Xu, 2016 (42)	Sarcoma	Kunming	M	NR	24g-26g	S180	CV17,CV12,CV6	SM	1/d,2w	Serum	CBA	TV,TW,spleen index,TNF-α,IL-2,IL-6,IFN-γ,TIR
24	Pei Wang, 2016 (43)	HCC	Wistar	M	NR	180-200g	(Intraperitoneal injection of Diethylnitrosamine)	BL18	CM	1/2d,20d	Serum	ELISA	TNF-α
25	Wenjuan Huang, 2015 (44)	LC	C57BL/6J	M	6-8W	18-22g	Lewis	ST36	CM	1/d,10d	Serum	ELISA	TV,IL-2
26	Haiyan Li, 2012 (45)	HCC	BALB/C	M	6-8W	20 ± 2g	H_22_	DU14	CM	6 times	NR	NR	TW,spleen index,TIR
27	Linna Yu, 2011 (46)	HCC	BALB/C	M	6-8W	20 ± 2g	H_22_	DU14	CM	6 times	NR	NR	TW,TIR
28	Jian Pei, 2010 (47)	HCC	BALB/C	M	6-8W	20 ± 2g	H_22_	DU14	CM	1/2d,6 times	The cerebral cortex	ISH	IL-2
29	Nanling Li, 2005 (48)	LC	C57BL/6J	M/F	7-8W	17-21g	Lewis	BL23	MM	11 times	Serum	RIA	TW,IL-2,TIR
30	Haiteng Shuiye, 2005 (49)	CC	BALB/C	NR	3W	16 ± 2g	C-26	GV4	CM	1/d,17d	Serum	ELISA	TW,IL-2,TIR
31	Xuewu Li, 2005 (50)	LC	C57BL/6J	M/F	NR	17-21g	Lewis	BL23	CaM	1/d,8d	NR	NR	TW,TIR
32	Xingsheng Qiu, 2004 (51)	HCC	Kunming	NR	6-8W	18-22g	H_22_	CV8	CM	1/2d,6 times	Serum	ELISA	IL-2
33	Huihai Xiong, 2003 (52)	Sarcoma	Kunming	F	4-6W	18-22g	S180	CV4	SM	1/d,10d	Serum	ELISA	spleen index,IL-2
34	Bing Liu, 2003 (53)	Sarcoma	Kunming	F	4W	18-22g	S180	CV4	SM	1/d,10d	NR	NR	TW,TIR
35	Ke Jiang, 2003 (54)	HCC	NIH	M/F	NR	18-20g	H_22_	ST36,DU14	SM	1/d,10d	NR	NR	TW,TIR
36	Hai Wei, 2002 (55)	HCC	BALB/C	M	6-8W	20 ± 2g	H_22_	DU14	CM	1/2d,6 times	Spleen	ELISA	IL-2
37	Ping Wu, 2002 (56)	Sarcoma	Kunming	M/F	6-8W	18-22g	S180	CV4	CM	1/d,10d	Serum	MTT	IL-2
38	Jingyi Fu, 2001 (57)	Sarcoma	BALB/C	M/F	NR	22 ± 1g	S37	DU14,houhai	CM	1/d,10d	NR	NR	TW,TIR
39	Cui Han, 2001 (58)	Sarcoma	Kunming	F	NR	20–22 g	S180	DU14	MM	1/2d,5 times	NR	NR	TW,spleen index,TIR
40	Zhixin Yang, 2001 (59)	LYM	C57BL/6	M	6-8W	20 ± 2g	EL4	DU14	CM	1/2d,7 times	Macrophages	ELISA	TW,TNF-α,IL-6,TIR
41	Benqiang Rao, 2000 (60)	CRC	BALB/C	M/F	5-7W	20 ± 2g	SW-480	CV4	CM	1/d,15d	NR	NR	TW,TIR
42	Peifeng Chen, 1999 (61)	LYM	Wistar	M	NR	230-260g	yac-1	CV4	EM	1/d,10d	NR	NR	TW,TIR
43	Xuexin Wang, 1999 (62)	HCC	Kunming	M	NR	18-20g	H_22_	LR14,GB34,CV6	SM	1/d,11d	NR	NR	TW,TIR
44	Zhaoliang Tang, 1999 (63)	Sarcoma	Kunming	F	8-12W	18-22g	S180	CV4	SM	1/d,7d	Macrophages	3H-TdR	TW,spleen index,IL-2,TIR
45	Jian Pei, 1997 (64)	HCC	BALB/C	M/F	6-8W	NR	H_22_	DU14	CM	1/2d,6 times	Spleen	3H-TdR	IL-2
46	Jian Pei, 1996 (65)	Sarcoma	NIH	M/F	NR	20 ± 2g	S180	DU14	CM	1/2d,6 times	NR	NR	TW
47	Youmi Yang-1, 1989 (66)	Sarcoma	Kunming	M	NR	21-23g	S180	DU14	CM	1/2d,7 times	NR	NR	TW,TIR
48	Youmi Yang-2, 1989 (66)	Sarcoma	Kunming	M	NR	21-23g	S180	DU14	CM	1/2d,7 times	NR	NR	TW,TIR

HCC, Hepatocellular Carcinoma; LC, Lung Cancer; GC, Gastric cancer; CRC, Colorectal Cancer; CC, Colon Cancer; RC, Rectal Cancer; BC, breast cancer; LYM, Lymphoma;.

M, male; F, female; M/F, both of male and female; NR, not reported; w, week; d, day;

DU14, Dazhui; ST25, Tianshu; ST36, Zusanli; SP6, Sanyinjia; CV4, Guanyuan; CV6, Qihai; CV8, Shenque; CV12, Zhongwan; CV17, Danzhong; BL18, Ganshu; BL20, Pishu; BL21, Weishu; BL23, Shenshu; GV4, Mingmen; GB34, Yanglingquan; LR14, Qimen; LU1, Zhongfu;

HPM, herb-partitioned moxibustion; CM, moxa-cone moxibustion; SM, moxa-stick moxibustion; LM, laser moxibustion; CaM, cantharides moxibustion; MM, medicinal moxibustion; EM, electro-moxibustion.

ELISA, Enzyme-Linked Immunosorbent Assay; CBA, Cytometric Bead Array; RIA, Radio Immuno Assay; MTT, 3-(4,5-dimethylthiazol-2-yl)-2,5-diphenyltetrazolium bromide; 3H-TdR, ³H-TdR incorporation assay; ISH, *in situ* hybridization.

TW, Tumor Weight; TIR, Tumor Inhibitory Rate; TV, tumor volume.

### 3.3 Bias risk and quality assessment

**Figure 2:**
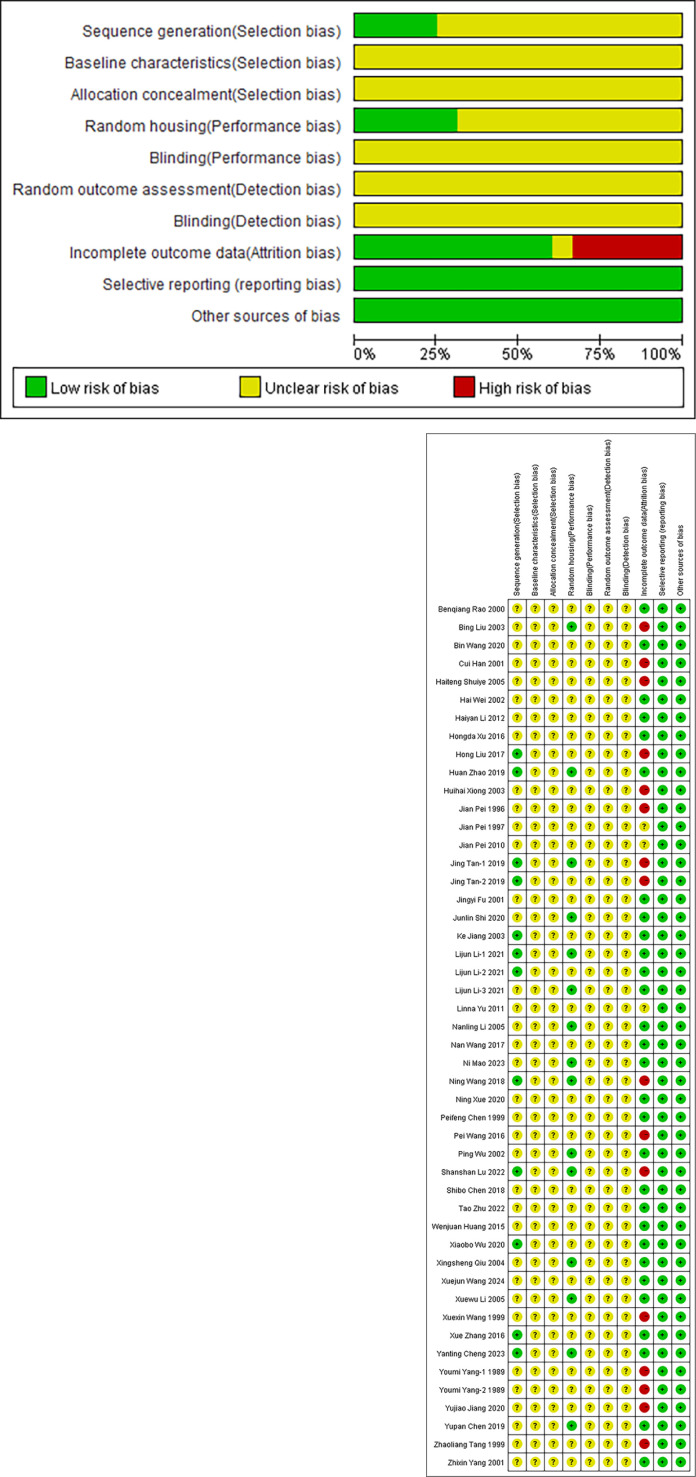
Quality assessment of the included trials risk of bias graph.

The quality assessment results of the 48 included studies are shown in **Figure 2**. It is worth noting that none of the studies reported specific information on the implementation of blinding for the animal experiment participants (such as researchers), due to the fact that all the included studies were animal experiments and it was difficult to effectively blind personnel in practical operation. According to the assessment results of the SYRCLE bias risk tool: (1) A total of 12 studies clearly described the generation method of the random sequence. 36 studies did not provide specific descriptions of their randomization methods. (2) All studies did not clearly report baseline data. (3) All studies did not clearly state the allocation concealment measures. (4) 15 studies described the same animal rearing conditions, while 33 studies did not provide such information. (5) All studies did not mention whether blinding was implemented in the same environment. (6) All studies did not clearly state the method for evaluating the results of the animals. (7) All studies did not clearly state whether the outcome assessors implemented blinding. (8) 16 studies were unable to assess the results of all animals due to the death of the animals during the measurement of the outcome indicators, but did not indicate whether the missing data affected the results. (9) All studies did not have selective result reporting. (10) The expected results of all studies have been reported, and there are no other sources of bias (**Figure 2**). Overall, the studies included in the meta-analysis are of medium methodological quality.

## 4 Meta-analysis results

### 4.1 The inhibitory effect of moxibustion on tumor growth

#### 4.1.1 Tumor volume suppression

**Figure 3:**
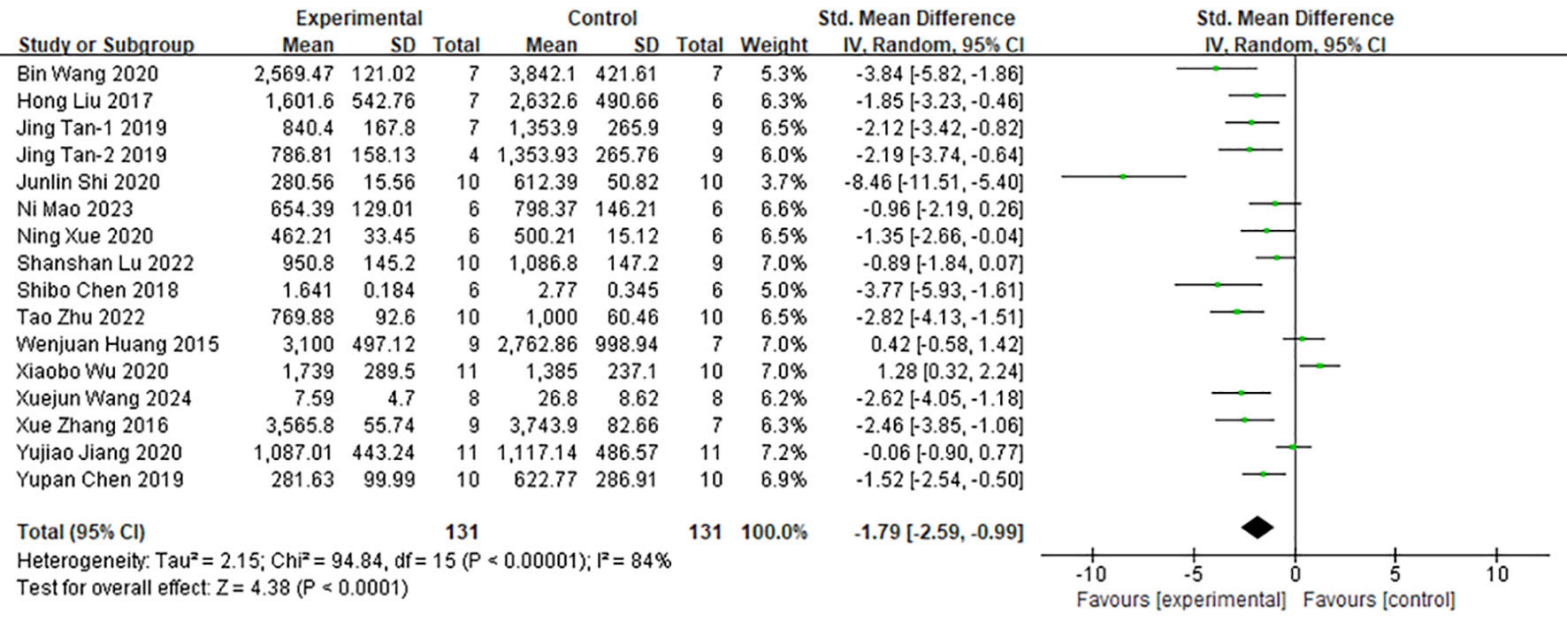
Comparison of tumor volume between the experimental group and the control group of tumor mice.

Sixteen of the 48 included studies reported the effects of moxibustion on tumor volume in animal models of cancer (**Figure 3**). Due to substantial heterogeneity across the studies (*I²* = 84%, *P* < 0.0001), a random-effects model was applied. Moxibustion showed a statistically significant inhibitory effect on tumor volume compared with the control group (n = 262; SMD = -1.79; 95% CI [-2.59, -0.99]). To explore potential sources of heterogeneity, subgroup analyses were conducted according to various study characteristics, as detailed in **Supplementary Table 1**. The type of moxibustion was identified as the primary contributor to heterogeneity. Among the four moxibustion methods—direct, indirect, suspended, and other techniques—indirect moxibustion exhibited the most pronounced reduction in tumor volume (SMD = -2.62; 95% CI [-4.05, -1.18]).

#### 4.1.2 Tumor weight suppression

**Figure 4:**
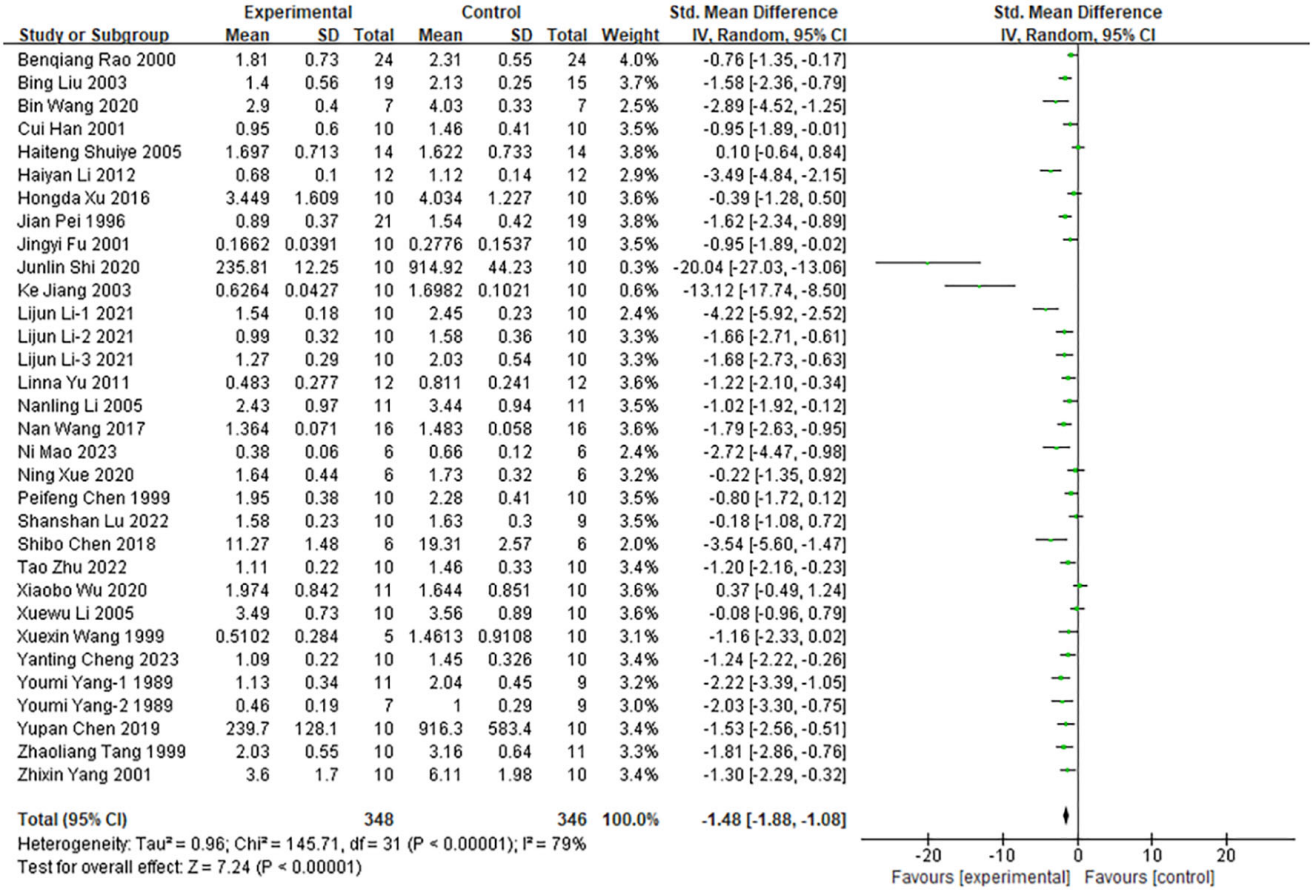
Comparison of tumor weight between the experimental group and the control group of tumor mice.

Of the 48 included studies, 32 reported the effect of moxibustion on tumor weight in animal models (**Figure 4**). Given the substantial heterogeneity across studies (*I²* = 79%, *P* < 0.00001), a random-effects model was used for the meta-analysis. Moxibustion demonstrated a statistically significant inhibitory effect on tumor weight compared with the control group (n = 694; SMD =–1.48; 95% CI [-1.88, –1.08]). To explore potential sources of heterogeneity, subgroup analyses were conducted according to various study characteristics, as shown in **Supplementary Table 2**. Animal species (*I²* = 55.9%), moxibustion method (*I²* = 80.0%), and cancer types (*I²* = 71.6%) were identified as major contributors to heterogeneity (all *P* < 0.05). In the subgroup analysis by animal species, heterogeneity was higher in studies using rats (*I²* = 93%) than in those using mice (*I²* = 76%), and the inhibitory effect of moxibustion on tumor weight was more pronounced in rat models (SMD=-3.98; 95% CI [-7.29, -0.67]). Subgroup analysis of moxibustion methods revealed significant differences in the inhibitory effects on tumor weight across the four approaches—direct moxibustion, indirect moxibustion, suspended moxibustion, and other techniques. Suspended moxibustion exhibited a stronger inhibitory effect compared to direct moxibustion (SMD = -2.31; 95% CI [-3.22, -1.40], *P* < 0.00001), whereas other techniques of moxibustion did not show a statistically significant effect (SMD = -0.48, *P* = 0.08). Furthermore, subgroup analysis by tumor type demonstrated that moxibustion exerted varying degrees of tumor weight reduction in models of liver cancer, lung cancer, gastric cancer, and S180 sarcoma, with the greatest effect observed in gastric cancer (SMD = -4.14; 95% CI [-7.20, -1.08]).

#### 4.1.3 Tumor inhibition rate

As shown in **Supplementary Table 3**, tumor inhibition rate was reported as an outcome measure in 26 of the 48 included studies, with values ranging from -4.62% to 65.09%. Among these, five studies reported tumor inhibition rates exceeding 50%, and half of them demonstrated antitumor effects greater than 38.91% following moxibustion treatment.

### 4.2 The regulation of immune organ function by moxibustion

#### 4.2.1 Spleen index

**Figure 5:**
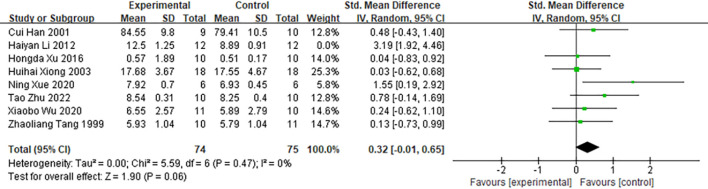
Comparison of spleen index between the experimental group and the control group of tumor-bearing mice.

Among the 48 included studies, eight studies reported spleen index as an outcome measure (**Figure 5**). Pooled analysis demonstrated that moxibustion increased spleen index levels in cancer animal models (n = 173; SMD = 0.69; 95% CI [0.08, 1.29]). Given that the heterogeneity across the studies exceeded 50% (*I²* = 71%, *P* < 0.05), a random-effects model was employed. As shown in **Supplementary Table 4**, subgroup analysis by animal species across the eight studies revealed that all studies employed mice as experimental animal models. Furthermore, the subgroup analyses indicated that mouse models (*I²* = 71%) and the use of direct moxibustion (*I²* = 78%) were the primary sources of heterogeneity, with an overall moderate to high level of heterogeneity observed across moxibustion methods (*I²* = 61.4%).

### 4.3 Regulation of immune factors by moxibustion

#### 4.3.1 Anti-tumor immune factors

##### 4.3.1.1 Interleukin-2

**Figure 6:**
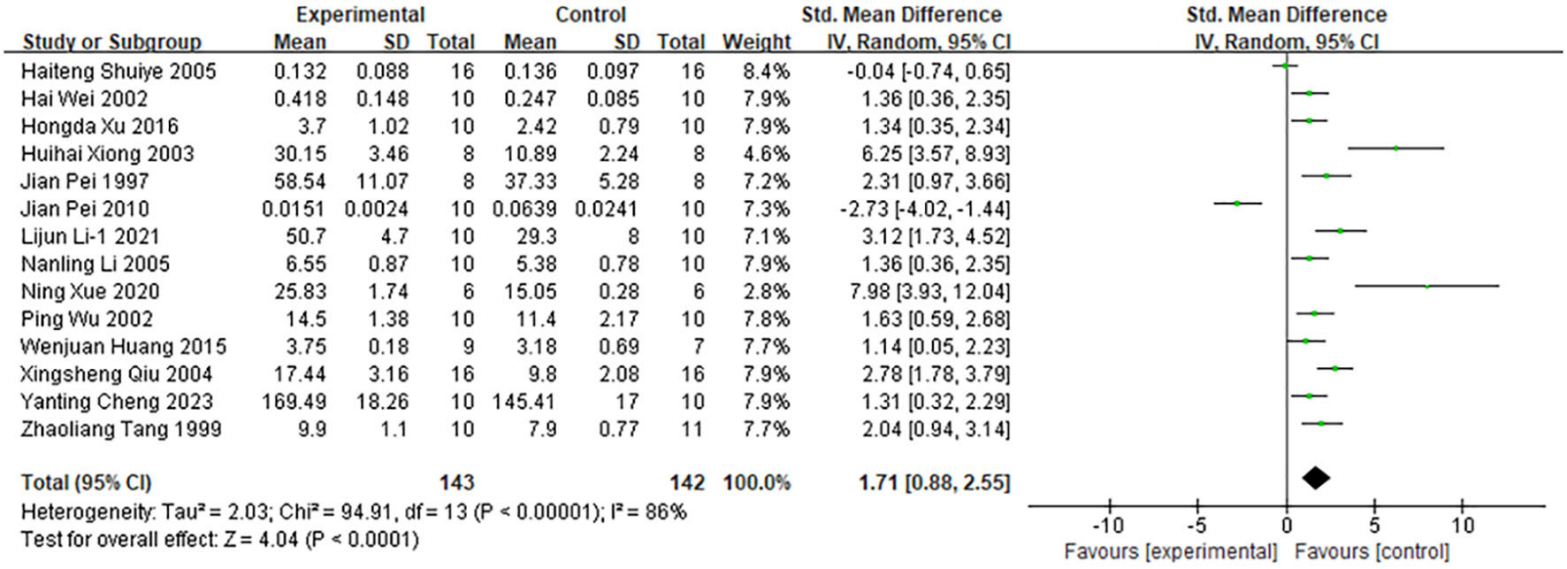
Comparison of interleukin-2 levels in tumor mice between the experimental group and the control group.

Among the 48 included studies, 14 reported interleukin-2 (IL-2) as an outcome measure (**Figure 6**). Pooled analysis showed that moxibustion significantly increased IL-2 levels in cancer animal models (n = 285; SMD = 1.71; 95% CI [0.88, 2.55]). Given the substantial heterogeneity across the studies (*I²* = 86%, *P* < 0.0001), a random-effects model was applied. As shown in **Supplementary Table 5** subgroup analysis revealed considerable heterogeneity by cancer types (*I²* = 85.4%). Further analysis stratified by cancer types indicated significant variation in the effect of moxibustion on IL-2 levels across different cancer types: hepatocellular carcinoma (SMD = 1.03; 95% CI [-0.69, 2.74]), lung cancer (SMD = 1.26; 95% CI [0.53, 1.99]), colon cancer (SMD = -0.04; 95% CI [-0.74, 0.65]), S180 sarcoma (SMD = 2.44; 95% CI [1.36, 3.52]), and breast cancer (SMD = 7.98; 95% CI [3.93, 12.04]). Liver cancer (*I²* = 92%) and S180 sarcoma (*I²* = 72%) were identified as the primary sources of heterogeneity.

##### 4.3.1.2 IFN-γ

**Figure 7:**
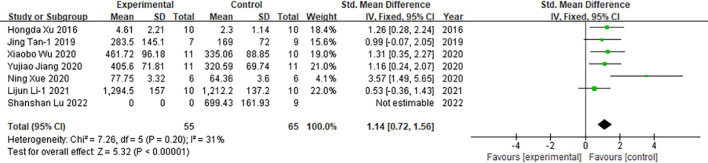
Comparison of interferon-γ in tumor mice between the experimental group and the control group.

Among the 48 included studies, seven studies reported interferon-γ(IFN-γ) as an outcome measure (**Figure 7**). Pooled analysis showed that moxibustion increased interferon-γ levels in cancer animal models (n = 120; SMD = 1.14; 95% CI [0.72, 1.56]). Given the low level of heterogeneity across studies (*I²* = 31%, *P* < 0.00001), a fixed-effect model was used for data synthesis.

##### 4.3.1.3 TNF-α

**Figure 8:**
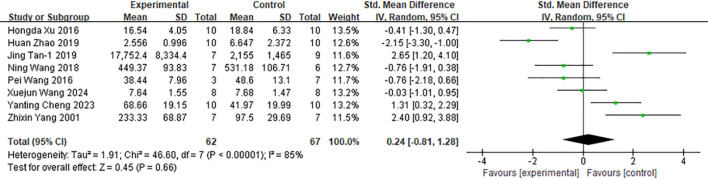
Comparison of tumor necrosis factor-α in tumor mice between the experimental group and the control group.

Among the 48 included studies, eight studies reported tumor necrosis factor-α (TNF-α) as an outcome measure (**Figure 8**). Pooled analysis demonstrated that moxibustion increased TNF-α levels in cancer animal models (n = 129; SMD = 0.24; 95% CI [−0.81, 1.28]). Given the substantial heterogeneity across studies (I² = 85%), a random-effects model was applied. As shown in **Supplementary Table 6**, subgroup analysis identified cancer types (*I²* = 62.2%) as the primary source of heterogeneity. In the subgroup analysis by tumor type, the regulatory effect of moxibustion on TNF-α demonstrated significant differences. In models of gastric cancer and lymphoma, moxibustion promoted TNF-α expression, whereas in S180 sarcoma, rectal cancer, and liver cancer models, it exerted an inhibitory effect on TNF-α levels.

#### 4.3.2 Pro-inflammatory factor

##### 4.3.2.1 IL-6

**Figure 9:**
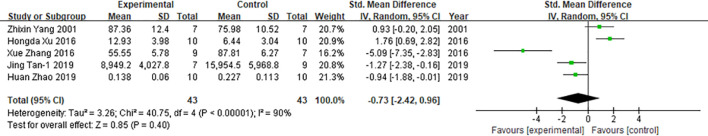
Comparison of interleukin-6 levels in tumor mice between the experimental group and the control group.

Among the 48 included studies, five studies reported interleukin-6 (IL-6) as an outcome measure(**Figure 9**). The overall analysis showed that moxibustion reduced IL-6 levels in cancer animal models (n = 86; SMD = -0.73; 95% CI [-2.42, 0.96]), although this effect was not statistically significant due to the confidence interval encompassing zero. Given the substantial heterogeneity across studies (*I²* = 90%), a random-effects model was applied for data synthesis, as detailed in **Supplementary Table 7**. Subgroup analysis identified duration of treatment (*I²* = 93.1%) and cancer types (*I²* = 92.6%) as the primary sources of heterogeneity. In the treatment duration subgroup, moxibustion exhibited a slight elevating effect on IL-6 in studies with a treatment course of ≥14 days (SMD = 0.11; 95% CI [-1.32, 1.54]), whereas it produced a pronounced inhibitory effect in those with a duration of <14 days (SMD = -5.09; 95% CI [-7.35, -2.83]). In subgroup analyses by cancer types, significant differences were observed in the regulatory effects of moxibustion on IL-6. Moxibustion increased IL-6 expression in S180 sarcoma (SMD = 1.76; 95% CI [0.69, 2.82]) and lymphoma models (SMD = 0.93; 95% CI [-0.20, 2.05]), whereas it reduced IL-6 levels in lung cancer and gastric cancer models, with the most pronounced inhibitory effect observed in lung cancer (SMD = -5.09; 95% CI [-7.35, -2.83]).

### 4.4 Sensitive analysis

**Figure 10:**
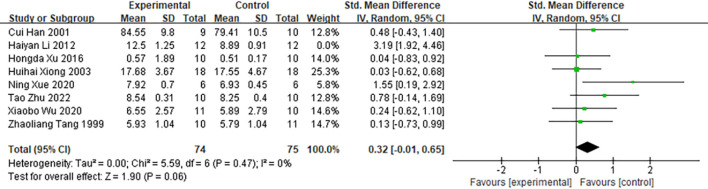
Comparison of spleen indices of tumor-bearing mice between the experimental group and the control group.

To evaluate the robustness of the pooled results, a sensitivity analysis was conducted by sequentially excluding individual studies and recalculating the effect estimates. The results indicated that the overall effect size changed significantly upon exclusion of the study by “Haiyan Li (2012) (45)”.(**Figure 10**). Prior to exclusion, the pooled effect size was SMD = 0.69; 95% CI [0.08, 1.29], with substantial heterogeneity (*I²* = 71%). Following exclusion of the study by “Haiyan Li (2012)”, the pooled effect size decreased to SMD = 0.32; 95% CI [−0.01, 0.65], and heterogeneity was eliminated (*I²* = 0%). These findings indicate that the overall result is sensitive to the inclusion of this particular study. While this meta-analysis suggests that moxibustion may have a positive effect on the spleen index, the sensitivity analysis implies that the robustness of this conclusion is limited. Following the exclusion of the study by “Haiyan Li (2012)”, both the point estimate of the effect size and its confidence interval exhibited substantial changes, and heterogeneity decreased markedly. This indicates that this study may constitute the primary source of heterogeneity and exerts considerable influence on the pooled effect estimate. It is plausible that this stems from the use of a hepatocellular carcinoma animal model and an intervention duration of less than 14 days in this study. Previous subgroup analyses also identified these two factors—specifically, the hepatocellular carcinoma model (*I²* = 80%) and intervention duration < 14 days (*I²* = 89%)—as major contributors to high heterogeneity. Therefore, the robustness of the current evidence on moxibustion’s effect on increasing the spleen index is notably influenced by individual studies. Future studies should aim to further validate the moderating effects of the hepatocellular carcinoma model and intervention duration on the efficacy of moxibustion, thereby strengthening the robustness of these findings.

### 4.5 Publication bias

**Figure 11:**
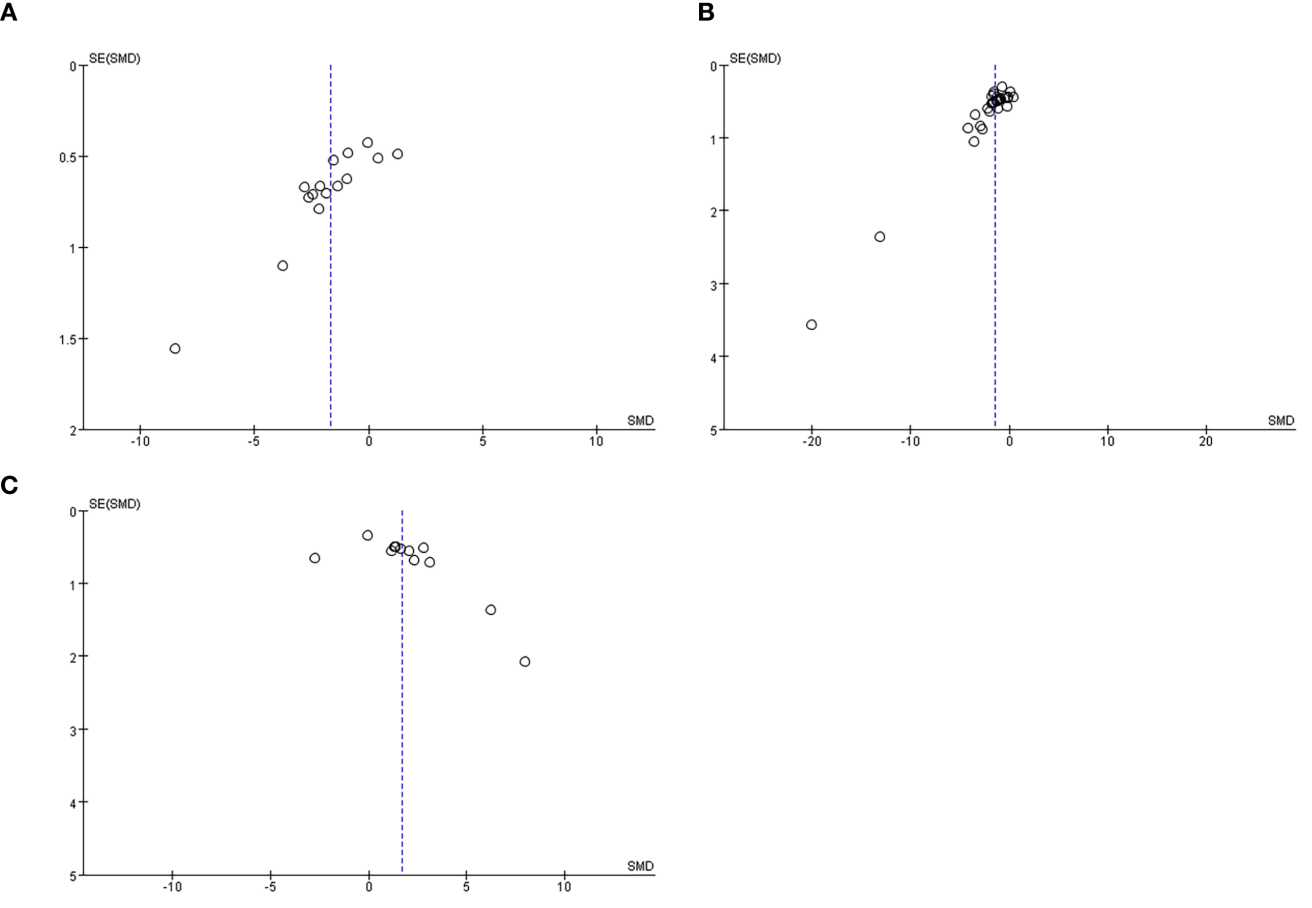
**(A)** A Funnel plot for the evaluation of publication bias for tumor volume. **(B)** Funnel plot for the evaluation of publication bias for tumor weight. **(C)** Funnel plot for the evaluation of publication bias for IL-2.

**Figure 11** presents the assessment of publication bias for tumor volume, tumor weight, and IL-2 levels. Funnel plots were used to evaluate potential bias. As shown in the figure, the funnel plots display evident asymmetry, suggesting the possible presence of publication bias. Additionally, Egger’s regression test confirmed statistically significant publication bias for all three outcomes: tumor volume, tumor weight, and IL-2 levels.

## 5 Discussion

Moxibustion is a traditional Chinese medical therapy that stimulates specific acupoints and meridians through the thermal effect, infrared radiation, photobiochemical effect, and bioelectric effect generated by burning mugwort, so as to dredge the meridians, promote blood circulation, and regulate the neuroendocrine immune function of the human body (67). Moxibustion can alleviate gastrointestinal reactions (68, 69), bone marrow suppression (70), cancer pain (71), cancer-related fatigue (72), etc. caused by cancer and cancer treatment. After analyzing the 48 included studies, it was found that moxibustion could increase the spleen index of cancer animal models, reduce tumor volume and weight, enhance the levels of anti-tumor immune factors IL-2, IFN-γ, and TNF-α in cancer organisms, and lower the level of pro-inflammatory immune factor IL-6.

### 5.1 Moxibustion enhances the spleen index and suppresses tumor growth in animal models of neoplasms

The research results show that the moxibustion therapy can effectively increase the tumor suppression rate in cancer animal models, and inhibit the growth of tumor volume and weight. As the largest immune organ and lymphoid organ, the spleen plays an indispensable role in regulating local and systemic immune responses (73). The spleen index (the ratio of spleen weight to body weight) can indirectly reflect the immune function of the human body. The meta-analysis demonstrates that moxibustion therapy consistently elevates the spleen index across diverse animal tumor models, with this effect remaining robust irrespective of treatment duration, animal species, cancer type, or moxibustion modality. Subgroup analyses identified three primary sources of heterogeneity: hepatocellular carcinoma (HCC) as the tumor model, direct moxibustion as the intervention method, and treatment duration <14 days. Notably, the greatest increase in spleen index was observed specifically under the combined conditions of HCC modeling, direct moxibustion application, and treatment duration <14 days.

Studies have shown that moxibustion at the “Shenque” acupoint can reduce cellular inflammatory necrosis by inhibiting the NF-κB/NLRP3/caspase-1 pathway, alleviate the inhibition of the spleen’s immune function by inflammation, improve the central arterial lumen structure of the splenic corpuscles and periarteriolar lymphatic sheaths in rats with exercise-induced fatigue, make the splenic cords arranged neatly, and distribute the splenic sinuses evenly (73). Another study has shown that moxibustion with herbal cakes at the acupoints of “Guanyuan”, “Shenque”, “Zhongwan” and “Zusanli” can improve the basic structure of the spleen tissue damaged by cyclophosphamide, increase the area of the marginal zone of the spleen tissue, increase the number of lymphocytes in the lymphoid sheath around the small central arteries of the spleen, and increase the number of CD4^+^T and CD8^+^T cells in the spleen tissue, thereby enhancing the immune function of the spleen in the body (74). Therefore, moxibustion may exert its anti-tumor immune effect by improving the tissue structure of the spleen, promoting the proliferation of T cells, and enhancing the immune function of the spleen.

### 5.2 Moxibustion increases levels of antitumor immune factors

#### 5.2.1 IL-2

IL-2 is mainly produced by activated T cells (75), and it may be one of the important influencing factors for moxibustion to exert its anti-tumor immune mechanism. Studies have shown that IL-2 can bind to the multimeric IL-2R, activate the signal transduction pathway, and promote the proliferation and activation of cytotoxic T cells and NK cells, thereby promoting the body to produce anti-tumor immunity (76). When cells become cancerous, PD-1 can bind to its ligand PD-L1 to mediate co-inhibitory signals for T cell activation, inhibiting the killing ability of tumor-infiltrating CD8^+^ T cells and thereby promoting the immune escape of tumor cells (77). Studies in Rheumatoid Arthritis (RA) rats further confirmed that the anti-inflammatory effect of moxibustion would be weakened after PD-1 was knocked down by gene (78). Therefore, we reasonably speculate that moxibustion may play a therapeutic role in diseases by affecting the PD1/PD-L1 axis. Moreover, studies have confirmed that in a mouse model bearing breast cancer tumors, moxibustion can reduce the protein expression levels of PD-1 and PD-L1 in tumor tissues and enhance the therapeutic effect on tumors (79). Anti-PD-1 immunotherapy relies on IL-2 signaling (80). Therefore, moxibustion may exert an anti-tumor effect by increasing the level of IL-2 and inhibiting the expression of PD-1 and PD-L1. However, in our study, we found that IL-2 exhibited high heterogeneity. Therefore, we conducted subgroup analysis to explore the sources of this heterogeneity. The analysis results showed that hepatocellular carcinoma and S180 sarcoma in the tumor models, as well as direct moxibustion and suspended moxibustion in the moxibustion methods, were the main factors leading to high heterogeneity. That is, the improvement of IL-2 levels by moxibustion may lead to high heterogeneity due to different tumor models and moxibustion methods.

#### 5.2.2 IFN-γ

IFN-γ is mainly produced by cytotoxic T lymphocytes, Th1 cells, and NK cells. IFN-γ exerts its anti-tumor effects mainly through two core ways: directly acting on tumor cells and regulating the functions of immune cells. In terms of direct action, IFN-γ directly induces programmed death of tumor cells by activating the JAK-STAT1 signaling pathway and upregulating the expression of apoptosis-related molecules such as Caspase-3 and Caspase-7 (81); In terms of immune regulation, in the tumor microenvironment, IFN-γcan increase the number of iNOS^+^CD206-M1 macrophages, thereby inhibiting tumor growth. Inducible nitric oxide synthase (iNOS) can stimulate the recruitment of T cells to tumor tissues, thus exerting anti-tumor immunity. Meanwhile, iNOS has been proven to inhibit the production of immunosuppressive and tumor growth factors (82, 83); Meanwhile, IFN-γ can also drive the maturation of dendritic cells, upregulate the secretion of their co-stimulatory molecules (such as CD80, CD86, and CCR7) and cytokines (such as IL-12), and effectively activate CD4^+^ and CD8^+^T cells (84, 85).

Moreover, studies have shown that herb cake-partitioned moxibustion may improve the immune function of immunosuppressed rabbits through the JAK2/STAT3 pathway mediated by IL-10 (86). Direct moxibustion can increase the expression of costimulatory molecules CD80 and CD86 on antigen-presenting cells (APCs) in the gastric mucosal tissue of gastric cancer model rats and the content of IFN-γin the serum, thereby promoting the activation of T lymphocytes and generating an immune response (87). Moxibustion with wheat-grain moxa can enhance the expression of granulocyte macrophage-colony stimulating factor (GM-CSF) in mice after cyclophosphamide chemotherapy, thereby increasing the activity of macrophages, enhancing their phagocytosis, and improving their antigen-presenting ability (88).

Therefore, moxibustion may directly induce apoptosis by increasing IFN-γ and activating the JAK-STAT signaling pathway; enhance the phagocytic ability of macrophages against tumor cells by increasing their activity and quantity; and increase the activation of T cells by increasing the expression of co-stimulatory factors on antigen-presenting cells, thereby exerting an anti-tumor immune response.

#### 5.2.3 TNF-α

TNF-α exerts dual effects on cancer (89). TNF-α is produced by most immune cells, including macrophages, neutrophils, fibroblasts, keratinocytes, NK cells, T cells, and B cells. TNF-α activates cells such as macrophages, dendritic cells, natural killer cells and T lymphocytes, enhancing the anti-tumor immune response (90). After TNF-α binds to tumor necrosis factor receptor-1, it can activate the Caspase enzyme family and promote apoptosis of tumor cells (91). On the other hand, TNF-α plays a pro-tumorigenic role in cancer. It can activate signaling pathways such as NF-κB and MAPK, promote the proliferation and survival of various cancer cells including breast cancer cells. It can also induce epithelial-mesenchymal transition, enhance the cell migration and invasion abilities, and thus promote metastasis (92, 93).

From the results of the Meta-analysis, the regulation of TNF-α levels in tumor models by moxibustion therapy presents multi-dimensional differential characteristics. Overall, moxibustion shows a trend of increasing TNF-α levels. Subgroup analysis reveals significant heterogeneity: regardless of the length of the treatment cycle, moxibustion can increase TNF-α levels. However, in terms of animal model types, moxibustion shows an inhibitory effect on TNF-α in rat cancer models, while it shows a promoting effect on TNF-α in mouse cancer models. Regarding tumor types, moxibustion significantly promotes the expression of TNF-α in gastric cancer and lymphoma models, but shows an inhibitory effect in S180 sarcoma, rectal cancer, and liver cancer models. In terms of moxibustion operation, direct moxibustion can increase TNF-α levels, while suspension moxibustion shows a decreasing effect on TNF-α levels.

In gastric cancer and lymphoma, moxibustion may exert anti-cancer effects by enhancing the Th1 response, increasing the activity of cytotoxic T cells, and up-regulating pro-inflammatory factors (TNF-α, IFN-γ) (7, 94). In the S180 sarcoma, rectal cancer, and liver cancer models, this intervention strategy may significantly reduce the expression of key inflammatory and oxidative stress regulatory factors such as TNF-α and ultimately exert anti-tumor effects by weakening the immunosuppressive function mediated by Treg cells, synchronously upregulating the expression levels of BRG1, Nrf2, and HO-1, and inhibiting the activation of the NF-κB signaling pathway (14, 21, 43, 95), thereby achieving an anti-cancer effect.

The increase in TNF-α levels caused by direct moxibustion may result from the strong local inflammatory or immune-stimulating effects it triggers. The early upregulation of TNF-α may reflect the acute immune activation stage, and subsequently, it can inhibit tumor growth by promoting anti-tumor immune responses (43, 95). The suspension moxibustion inhibits the level of TNF-α. This might be due to the gentle nature of the moxibustion method, with lower intensity of thermal stimulation and inflammatory stimulation. Thus, it exerts a profound regulatory effect on pro-inflammatory factors such as TNF-α, especially in chronic inflammatory or cancer models (96, 97).

Studies have shown that herb cake-partitioned moxibustion can improve the peripheral immunosuppressive state of rats by reducing the expression of NF-κB and IL-18 in brain tissue and increasing the level of TNF-α in serum (98). Moxibustion serum significantly enhances the cytotoxicity of cytotoxic T lymphocytes (CTL) in the index growth phase tumor-infiltrating lymphocytes (TIL), significantly increases the content of TNF-α in the supernatant of TIL culture, and to some extent enhances the level of IFN-γ produced by TIL, thereby promoting the specific killing activity of TIL against tumor cells (99). Another study has shown that moxibustion with seed-sized moxa cone can significantly increase the levels of IL-2 and TNF-α in the serum of mice with liver cancer tumors, as well as the expression of Caspase-3, Caspase-9 proteins and their mRNA in the tumor tissues (22).

Therefore, moxibustion may exert its anti-tumor effect by increasing the level of TNF-α, enhancing the cytotoxicity of T lymphocytes, and boosting the anti-tumor immune response; by activating the expression of Caspase enzymes, promoting tumor cell apoptosis, thereby achieving the anti-tumor effect.

### 5.3 Suppression of pro-inflammatory immune factors by moxibustion

#### 5.3.1 IL-6

IL-6 is a pro-inflammatory cytokine that participates in immune regulation. It is secreted by various cells in the tumor microenvironment, including dendritic cells, macrophages, T lymphocytes, B lymphocytes, and cancer cells. IL-6 can influence various aspects of tumor development by regulating proliferation, apoptosis, metabolism, survival, angiogenesis, and metastasis (100). Numerous studies have shown that the IL - 6/JAK2/STAT3 signaling pathway is abnormally and highly active in various cancers, such as gastric cancer, breast cancer, liver cancer, colorectal cancer, colon cancer, ovarian cancer, lung cancer, and pancreatic cancer. It strongly inhibits the anti - tumor immune response (101).

The results of the meta-analysis showed that moxibustion inhibited the secretion of IL-6 in tumor models. According to the subgroup analysis, the regulatory effect of moxibustion on IL-6 levels exhibited multi-dimensional differences in terms of time, species, tumor model, and moxibustion method. Overall, moxibustion tended to inhibit IL-6 levels, but there was significant heterogeneity in different subgroup analyses. Regarding the treatment time, when less than 14 days, the inhibitory effect of moxibustion on IL-6 was significant; when greater than or equal to 14 days, it showed a slight increase in IL-6 levels. The animal species subgroup all showed inhibitory effects, with mice showing a more significant inhibitory effect. In different cancer models, in lung cancer and gastric cancer models, moxibustion significantly reduced IL-6 levels, while in S180 sarcoma and lymphoma models, moxibustion instead promoted IL-6 expression. Regardless of whether direct moxibustion or suspended moxibustion was used, it all showed an inhibitory effect on IL-6 levels.

Studies have shown that short-term intervention measures, such as acute exercise or brief pharmacological blockade, can temporarily inhibit or regulate the release of IL-6, usually providing immediate but transient anti-inflammatory or protective effects (102–104), However, these effects are usually difficult to sustain, and the IL-6 level may rebound after the intervention stops. In contrast, long-term intervention measures (such as continuous exercise training or long-term environmental exposure) can induce more persistent changes in IL-6 regulation, mainly by improving immune homeostasis and reducing chronic inflammation (103, 105, 106). Therefore, we believe that short-term moxibustion intervention can transiently inhibit the production of IL-6, while long-term intervention may slightly increase the level of IL-6 through immune homeostasis.

In different cancer models, moxibustion has different effects on IL-6. In lung cancer and gastric cancer models, moxibustion significantly reduces the level of IL-6. In S180 sarcoma and lymphoma models, moxibustion promotes the expression of IL-6 instead. This immunomodulatory effect may be related to the difference in epithelial tumor models. Recent studies have shown that the inflammatory state of the tumor microenvironment (TME) (including factors such as IL-6 levels) can vary significantly depending on the type of tumor model used. Among them, the *in situ* (primary) model is more capable of simulating the natural environment of the tumor (107–109), while the subcutaneous (ectopic) transplantation model cannot. Multiple studies have shown that *in situ* models typically exhibit higher levels of immune cell infiltration, stronger expression of pro-inflammatory cytokines (including IL-6), as well as more immunosuppressive or complex immune environments (110–112). Epithelial tumors (lung cancer, gastric cancer, colorectal cancer) usually exhibit tumor-promoting inflammation driven by IL-6. In this case, inhibiting IL-6 is beneficial (7, 15, 113–115). In contrast, sarcomas and lymphomas may have different cytokine profiles and immune cell compositions, in which the upregulation of IL-6 may reflect a shift towards anti-tumor immunity or a compensatory response (14, 116–118). That is to say, promoting the expression of IL-6 is beneficial in this situation.

Studies have shown that moxibustion with seed-sized moxa cone can reduce the levels of IL-6 and TNF-α in the serum of mice with ulcerative colitis models, decrease the expression of JAK2 and STAT3 mRNA and protein in the colonic tissue, and improve the damage of the colonic mucosa in ulcerative colitis model mice (119). Moxibustion with seed-sized moxa cone can reduce the level of IL-6 in the serum and the expression level of STAT 3 in the tumor of Lewis lung cancer mice (120). The use of incense moxibustion can reduce the content of IL-6 in the serum of acute eczema rats and the expression levels of STAT3 and p-STAT3 proteins in the damaged skin tissues, thereby alleviating the inflammatory response in acute eczema rats (121). Therefore, moxibustion may exert an anti-tumor immune response by reducing the level of IL-6 and affecting the IL-6/JAK2/STAT3 signaling pathway.

## 6 Conclusion

Through Meta-analysis, we conclude that moxibustion exerts its anti-tumor effect through a multi-dimensional and synergistic immune regulation network. The mechanisms can be systematically summarized as follows:

First, moxibustion may alleviate inflammatory damage to immune organs (such as the spleen) by inhibiting inflammatory pathways such as NF-κB/NLRP3/caspase-1, and improve their tissue structure and function, providing a favorable microenvironment for the proliferation and activation of immune cells such as T cells. On this basis, moxibustion upregulates the levels of key cytokines IL-2, IFN-γ, and TNF-α: IL-2 promotes the proliferation and activation of cytotoxic T cells and NK cells; IFN-γ not only directly induces apoptosis of tumor cells through the JAK-STAT1 signaling pathway, but also drives the maturation of dendritic cells and enhances their antigen - presenting ability. At the same time, it promotes the polarization of macrophages into the M1 type and enhances their phagocytic function; TNF-α further enhances the killing activity of cytotoxic T cells and participates in inducing tumor cell death. In addition, moxibustion may enhance the immune response efficiency of T cells by inhibiting the PD-1/PD-L1 immune checkpoint axis and removing the immunosuppressive signals in the tumor microenvironment. These mechanisms are interrelated and act synergistically to jointly enhance immune surveillance, promote the clearance of tumor cells, and ultimately achieve multi-pathway anti-tumor immune effects.

The research findings demonstrate the beneficial effects of moxibustion therapy on tumor volume, tumor weight, and immune parameters across nine types of tumor animal models, including hepatocellular carcinoma, lung cancer, gastric cancer, sarcoma, breast cancer, colon cancer, rectal cancer, lymphoma, and colorectal cancer. The results confirm that moxibustion exerts an inhibitory effect on tumor growth, increases the spleen index, elevates levels of anti-tumor immune factors such as IL-2, IFN-γ, and TNF-α, suppresses the expression of the pro-inflammatory cytokine IL-6, and enhances overall immune function. Despite these findings, certain limitations remain in this study, and further in-depth research is needed to comprehensively evaluate the therapeutic potential of moxibustion in oncological applications.

## Data Availability

The original contributions presented in the study are included in the article/**Supplementary Material**. Further inquiries can be directed to the corresponding author.
