# Supplementary material for: Effects of moxibustion therapies on immune function in cancer animal models: a systematic review and meta-analysis
Source: Front Immunol. 2026 Mar 12;17:1724707. doi: 10.3389/fimmu.2026.1724707 (PMC13017855; doi:10.3389/fimmu.2026.1724707)
Supplement: Supplementary file 1 [file Table1.docx]

**Supplementary Tables**

**Supplementary Table 1** The subgroup analysis of Tumor volume

| **outcome** | **Subgroup** | **Studies** | **Statistical Method** | **Effect Estimate** | **I^2^ (%)** |
| --- | --- | --- | --- | --- | --- |
| **Tumor volume** | **Duration of treatment (day)** |  |  |  | 0% |
|  | ≥14d | 12 | Std. Mean Difference (IV, Random, 95% CI) | -1.87 [-2.84, -0.90] | 85% |
|  | ＜14d | 4 | Std. Mean Difference (IV, Random, 95% CI) | -1.63 [-3.25, -0.02] | 85% |
|  | **Animal species** |  |  |  | 58.4% |
|  | Rat | 4 | Std. Mean Difference (IV, Random, 95% CI) | -3.05 [-4.91, -1.19] | 83% |
|  | Mice | 12 | Std. Mean Difference (IV, Random, 95% CI) | -1.43 [-2.30, -0.56] | 83% |
|  | **Cancer types** |  |  |  | 44.1% |
|  | Lung cancer | 9 | Std. Mean Difference (IV, Random, 95% CI) | -1.15 [-2.14, 0.15] | 84% |
|  | Gastric cancer | 4 | Std. Mean Difference (IV, Random, 95% CI) | -3.05 [-4.91, -1.19] | 83% |
|  | Hepatocellular carcinoma | 1 | Std. Mean Difference (IV, Random, 95% CI) | -2.82 [-4.13, -1.51] |  |
|  | Rectal cancer | 1 | Std. Mean Difference (IV, Random, 95% CI) | -2.62 [-4.05, -1.18] |  |
|  | Breast cancer | 1 | Std. Mean Difference (IV, Random, 95% CI) | -1.35 [-2.66, -0.04] |  |
|  |  |  |  |  |  |
|  | **Moxibustion methods** |  |  |  | 79.3% |
|  | Direct moxibustion | 6 | Std. Mean Difference (IV, Random, 95% CI) | -1.85 [-3.08, -0.62] | 79% |
|  | Indirect moxibustion | 1 | Std. Mean Difference (IV, Random, 95% CI) | -2.62 [-4.05, -1.18] |  |
|  | Suspended moxibustion | 7 | Std. Mean Difference (IV, Random, 95% CI) | -2.36 [-3.51, -1.22] | 79% |
|  | Other moxibustion | 2 | Std. Mean Difference (IV, Random, 95% CI) | 0.59 [-0.73, 1.90] | 77% |
|  | Total | 16 | Std. Mean Difference (IV, Random, 95% CI) | -1.79 [-2.59, -0.99] | 84% |

**Supplementary Table 2** The subgroup analysis of Tumor weight

| **outcome** | **Subgroup** | **Studies** | **Statistical Method** | **Effect Estimate** | **I^2^ (%)** |
| --- | --- | --- | --- | --- | --- |
| **Tumor weight** | **Duration of treatment (day)** |  |  |  | 0% |
|  | ≥14d | 19 | Std. Mean Difference (IV, Random, 95% CI) | -1.51 [-2.08, -0.93] | 81% |
|  | ＜14d | 13 | Std. Mean Difference (IV, Random, 95% CI) | -1.45 [-2.01, -0.90] | 75% |
|  | **Animal species** |  |  |  | 55.9% |
|  | Rat | 3 | Std. Mean Difference (IV, Random, 95% CI) | -3.98 [-7.29, -0.67] | 93% |
|  | Mice | 29 | Std. Mean Difference (IV, Random, 95% CI) | -1.42[-1.80, -1.03] | 76% |
|  | **Cancer types** |  |  |  | 71.6% |
|  | Sarcoma | 12 | Std. Mean Difference (IV, Random, 95% CI) | -1.51 [-1.93, -1.09] | 53% |
|  | Hepatocellular carcinoma | 6 | Std. Mean Difference (IV, Random, 95% CI) | -2.29 [-3.59, -1.00] | 85% |
|  | Lung cancer | 7 | Std. Mean Difference (IV, Random, 95% CI) | -1.17 [-2.12, -0.23] | 79% |
|  | Gastric cancer | 3 | Std. Mean Difference (IV, Random, 95% CI) | -4.14 [-7.20, -1.08] | 92% |
|  | Breast cancer | 1 | Std. Mean Difference (IV, Random, 95% CI) | -0.22 [-1.35, 0.92] |  |
|  | Colon cancer | 1 | Std. Mean Difference (IV, Random, 95% CI) | 0.10 [-0.64, 0.84] |  |
|  | Lymphoma | 1 | Std. Mean Difference (IV, Random, 95% CI) | -1.30 [-2.29, -0.32] |  |
|  | Colorectal cancer | 1 | Std. Mean Difference (IV, Random, 95% CI) | -0.76 [-1.35, -0.17] |  |
|  |  |  |  |  |  |
|  | **Moxibustion methods** |  |  |  | 80.0% |
|  | Direct moxibustion | 13 | Std. Mean Difference (IV, Random, 95% CI) | -1.45 [-1.95, -0.96] | 69% |
|  | Indirect moxibustion | 1 | Std. Mean Difference (IV, Random, 95% CI) | -0.76 [-1.35, -0.17] |  |
|  | Suspended moxibustion | 13 | Std. Mean Difference (IV, Random, 95% CI) | -2.31 [-3.22, -1.40] | 85% |
|  | Other moxibustion | 5 | Std. Mean Difference (IV, Random, 95% CI) | -0.48 [-1.02, 0.06] | 45% |
|  | Total | 32 | Std. Mean Difference (IV, Random, 95% CI) | -1.48 [-1.88, -1.08] | 79% |

**Supplementary Table 3** Characteristics of Tumor、Intervention and Tumor Inhibitory rate.

| **Study** | **Tumor** | | | **Intervention** | | | | | | | **Tumor Inhibitory rate(%)** |
| --- | --- | --- | --- | --- | --- | --- | --- | --- | --- | --- | --- |
|  | **Tumor cell /Dose** | **Inoculation site** | **method** | **Moxi-**  **bustion point** | **Sample (T)** | **Sample (C)** | **Intervene Time**  **(day)** | **Frequency**  **(day/time) ×**  **times** | **Duration**  **(day)** | **Follow Time**  **(day)** |  |
| Benqiang Rao 2000 | SW-480  1×1×1mm^3^ | right axillary region | s.c. | Guanyuan | 24 | 24 | 0 | 1×15 | 15 | 16 | 21.70% |
| Bing Liu 2003 | S180  3.6×10^6^ | Left axilla | s.c. | Guanyuan | 20 | 20 | ＋1 | 1×10 | 10 | 10 | 34.00% |
| Cui Han 2001 | S180  2×10^6^ | right axillary region | s.c. | Dazhui | 10 | 10 | ＋1 | 2×5 | 10 | 11 | 38.52% |
| Haiteng Shuiye 2005 | C-26  1×10^6^ | plasma membrane of ceacum | i.p. | Mingmen | 15 | 15 | ＋1 | 1×17 | 17 | 18 | -4.62% |
| Haiyan Li 2012 | H22 1×10⁶ | left armpit | s.c. | Dazhui | 12 | 12 | ＋1 | 2×6 | 12 | 13 | 39.30% |
| Hongda Xu 2016 | S180  4×10⁶ | Left axillary region | s.c. | Danzhong、Zhongwan、Qihai | 10 | 10 | 0 | 1×14 | 14 | 14 | 13.69% |
| Jing Tan-1 2019 | Walker-256  0.5×0.5×0.5 cm^3^ | stomach | gastric transplantation | Zhongwan、Guanyuan、Zusanli、Pishu、Weishu | 7 | 9 | ＋7 | 1×21 | 21 | 21 | 37.93% |
| Jing Tan-2 2019 | Walker-256  0.8×0.8×0.5 cm^3^ | stomach | gastric transplantation | Zhongwan、Guanyuan、Zusanli、Pishu、Weishu | 4 | 9 | ＋7 | 1×21 | 21 | 21 | 41.89% |
| Jingyi Fu 2001 | S37  1×10^5^ | right axillary region | s.c. | Dazhui、Houhai | 10 | 10 | ＋0 | 1×10 | 10 | 11 | 40.12% |
| Junlin Shi 2020 | Walker-256  2×2×2mm^3^ | stomach | Ectopic transplantation of cancer tissue blocks | Zhongwan、Guanyuan、Zusanli、Pishu、Weishu | 10 | 10 | ＋7 | 1×14 | 14 | 14 | 54.78% |
| Ke Jiang 2003 | H22  NR | axillary region | s.c. | Zusanli、Dazhui | 10 | 10 | ＋4 | 1×10 | 10 | 10 | 63.11% |
| Linna Yu 2011 | H22  1×10⁶ | Left axillary region | s.c. | Dazhui | 12 | 12 | ＋1 | 2×6 | 12 | 13 | 40.70% |
| Nanling Li 2005 | Lewis  2×10⁶ | right axillary region | s.c. | Shenshu | 12 | 12 | ＋1 | 1×11 | 11 | 12 | 29.30% |
| Nan Wang 2017 | SGC-  7901  1×1×1 mm^3^ | **left hand groins** | s.c. | Zhongwan、Zusanli | 16 | 16 | ＋1 | 1×15 | 15 | 15 | 8.00% |
| Ni Mao 2023 | LLC  1×10^5^ | \| right flank \| \| --- \| | s.c. | Zusanli、Mingmen | 6 | 6 | ＋7 | 2×7 | 14 | 14 | 42.44% |
| Ning Xue 2020 | 4T1   4×10⁶ | 4^th^ left inguinal mammary fat pad | s.c. | Zusanli | 6 | 6 | NR | 1×14 | 14 | 15 | 5.60% |
| Peifeng Chen 1999 | yac-1  2×10⁶ | **right axillary region** | s.c. | Guanyuan | 10 | 10 | ＋1 | 1×10 | 10 | 11 | 14.47% |
| Shibo Chen 2018 | Lewis  1.0×10^6^ | **right armpit** | s.c. | Zhongfu | 6 | 6 | NR | 1×14 | 14 | 14 | 41.64% |
| Tao Zhu 2022 | Hepa1-6  6×10^5^ | right armpit | s.c. | Dazhui，Zusanli，Sanyinjiao | 10 | 10 | ＋5 | 1×10 | 10 | 11 | 23.90% |
| Xuewu Li 2005 | Lewis  2×10^9^ | **right axillary region** | s.c. | Shenshu | 10 | 10 | ＋1 | 1×8 | 8 | 9 | 1.74% |
| Youmi Yang-1 1989 | S180  NR | **right axillary region** | s.c. | Dazhui | 25 | 38 | ＋1 | 2×7 | 14 | 14 | 44.60% |
| Yupan Chen 2019 | Walker-256  5×5×5mm^3^ | stomach | tumor tissue transplantation | Zhongwan、Guanyuan、Zusanli、Pishu、Weishu | 10 | 10 | ＋7 | 1×14 | 14 | 15 | 54.78% |
| Zhixin Yang 2001 | EL4  1×10^6^ | **left hand groins** | s.c. | Dazhui | 10 | 10 | ＋1 | 2×7 | 14 | 24 | 41.08% |
| Xuexin Wang, 1999 | H  22  1×10  6 | right axillary region | s.c. | Qimen、Yanglingquan、Qihai | 5 | 10 | ＋3 | 1×11 | 11 | 12 | 65.09% |
| Youmi Yang-2, 1989 | S180  NR | **right axillary region** | s.c. | Dazhui | 25 | 38 | ＋1 | 2×7 | 14 | 14 | 54.00% |
| Zhaoliang Tang,1999 | S180  NR | **right axillary region** | s.c. | Guanyuan | 10 | 11 | ＋4 | 1×7 | 7 | 8 | 36.10% |

T: Treatment C: Control s.c.:Subcutaneous injection i.p.:Intraperitoneal injection i.v.:Intravenous ; NR: Not reported.

**Supplementary Table 4** The subgroup analysis of Spleen index

| **outcome** | **Subgroup** | **Studies** | **Statistical Method** | **Effect Estimate** | **I^2^ (%)** |
| --- | --- | --- | --- | --- | --- |
| **Spleen index** | **Duration of treatment (day)** |  |  |  | 0% |
|  | ≥14d | 3 | Std. Mean Difference (IV, Random, 95% CI) | 0.46 [-0.31, 1.22] | 43% |
|  | ＜14d | 5 | Std. Mean Difference (IV, Random, 95% CI) | 0.82 [-0.07, 1.72] | 80% |
|  | **Animal species** |  |  |  |  |
|  | Rat | 0 | Std. Mean Difference (IV, Random, 95% CI) | Not estimable |  |
|  | Mice | 8 | Std. Mean Difference (IV, Random, 95% CI) | 0.69 [0.08, 1.29] | 71% |
|  | **Cancer types** |  |  |  | 47.5% |
|  | Sarcoma | 4 | Std. Mean Difference (IV, Random, 95% CI) | 0.14 [-0.26, 0.54] | 0% |
|  | Hepatocellular carcinoma | 2 | Std. Mean Difference (IV, Random, 95% CI) | 1.94 [-0.42, 4.30] | 89% |
|  | Breast cancer | 1 | Std. Mean Difference (IV, Random, 95% CI) | 1.55 [0.19, 2.92] |  |
|  | Lung cancer | 1 | Std. Mean Difference (IV, Random, 95% CI) | 0.24 [-0.62, 1.10] |  |
|  | **Moxibustion methods** |  |  |  | 61.4% |
|  | Direct moxibustion | 3 | Std. Mean Difference (IV, Random, 95% CI) | 1.80 [0.35, 3.24] | 78% |
|  | Suspended moxibustion | 3 | Std. Mean Difference (IV, Random, 95% CI) | 0.06 [-0.39, 0.51] | 0% |
|  | Other moxibustion | 2 | Std. Mean Difference (IV, Random, 95% CI) | 0.35 [-0.28, 0.98] | 0% |
|  | Total | 8 | Std. Mean Difference (IV, Random, 95% CI) | 0.69 [0.08, 1.29] | 71% |

**Supplementary Table 5** The subgroup analysis of IL-2

| **outcome** | **Subgroup** | **Studies** | **Statistical Method** | **Effect Estimate** | **I^2^ (%)** |
| --- | --- | --- | --- | --- | --- |
| **IL-2** | **Duration of treatment (day)** |  |  |  | 0% |
|  | ≥14d | 6 | Std. Mean Difference (IV, Random, 95% CI) | 2.10 [0.80, 3.40] | 87% |
|  | ＜14d | 8 | Std. Mean Difference (IV, Random, 95% CI) | 1.46 [0.28, 2.65] | 87% |
|  | **Animal species** |  |  |  |  |
|  | Rat | 0 | Std. Mean Difference (IV, Random, 95% CI) | Not estimable |  |
|  | Mice | 14 | Std. Mean Difference (IV, Random, 95% CI) | 1.71 [0.88, 2.55] | 86% |
|  | **Cancer types** |  |  |  | 85.4% |
|  | Hepatocellular carcinoma | 5 | Std. Mean Difference (IV, Random, 95% CI) | 1.03 [-0.69, 2.74] | 92% |
|  | Sarcoma | 5 | Std. Mean Difference (IV, Random, 95% CI) | 2.44 [1.36, 3.52] | 72% |
|  | Lung cancer | 2 | Std. Mean Difference (IV, Random, 95% CI) | 1.26 [0.53, 1.99] | 0% |
|  | Colon cancer | 1 | Std. Mean Difference (IV, Random, 95% CI) | -0.04 [-0.74, 0.65] |  |
|  | Breast cancer | 1 | Std. Mean Difference (IV, Random, 95% CI) | 7.98 [3.93, 12.04] |  |
|  |  |  |  |  |  |
|  | **Moxibustion methods** |  |  |  | 34.2% |
|  | Direct moxibustion | 9 | Std. Mean Difference (IV, Random, 95% CI) | 1.31 [0.21, 2.41] | 89% |
|  | Suspended moxibustion | 4 | Std. Mean Difference (IV, Random, 95% CI) | 2.78 [1.33, 4.23] | 78% |
|  | Other moxibustion | 1 | Std. Mean Difference (IV, Random, 95% CI) | 1.36 [0.36, 2.35] |  |
|  | Total | 14 | Std. Mean Difference (IV, Random, 95% CI) | 1.71 [0.88, 2.55] | 86% |

**Supplementary Table 6** The subgroup analysis of TNF-α

| **outcome** | **Subgroup** | **Studies** | **Statistical Method** | **Effect Estimate** | **I^2^ (%)** |
| --- | --- | --- | --- | --- | --- |
| **TNF-α** | **Duration of treatment (day)** |  |  |  | 46.7% |
|  | ≥14d | 7 | Std. Mean Difference (IV, Random, 95% CI) | 0.08 [-1.07, 1.22] | 87% |
|  | ＜14d | 1 | Std. Mean Difference (IV, Random, 95% CI) | 1.31 [0.32, 2.29] |  |
|  | **Animal species** |  |  |  | 7.2% |
|  | Rat | 4 | Std. Mean Difference (IV, Random, 95% CI) | -0.29 [-2.19, 1.61] | 89% |
|  | Mice | 4 | Std. Mean Difference (IV, Random, 95% CI) | 0.72 [-0.42, 1.86] | 79% |
|  | **Cancer types** |  |  |  | 62.2% |
|  | Hepatocellular carcinoma | 3 | Std. Mean Difference (IV, Random, 95% CI) | -0.02 [-1.48, 1.44] | 78% |
|  | Gastric cancer | 2 | Std. Mean Difference (IV, Random, 95% CI) | 0.23 [-4.48, 4.93] | 96% |
|  | Lymphoma | 1 | Std. Mean Difference (IV, Random, 95% CI) | 2.40 [0.92, 3.88] |  |
|  | Rectal cancer | 1 | Std. Mean Difference (IV, Random, 95% CI) | -0.03[-1.01, 0.95] |  |
|  | Sarcoma | 1 | Std. Mean Difference (IV, Random, 95% CI) | -0.41 [-1.30, 0.47] |  |
|  |  |  |  |  |  |
|  | **Moxibustion methods** |  |  |  | 0% |
|  | Direct moxibustion | 4 | Std. Mean Difference (IV, Random, 95% CI) | 0.53 [-0.92, 1.99] | 82% |
|  | Indirect moxibustion | 1 | Std. Mean Difference (IV, Random, 95% CI) | -0.03[-1.01, 0.95] |  |
|  | Suspended moxibustion | 3 | Std. Mean Difference (IV, Random, 95% CI) | -0.02 [-2.39, 2.36] | 92% |
|  | Total | 8 | Std. Mean Difference (IV, Random, 95% CI) | 0.24 [-0.81, 1.28] | 85% |

**Supplementary Table 7** The subgroup analysis of IL-6

| **outcome** | **Subgroup** | **Studies** | **Statistical Method** | **Effect Estimate** | **I^2^ (%)** |
| --- | --- | --- | --- | --- | --- |
| **IL-6** | **Duration of treatment (day)** |  |  |  | 93.1% |
|  | ≥14d | 4 | Std. Mean Difference (IV, Random, 95% CI) | 0.11 [-1.32, 1.54] | 86% |
|  | ＜14d | 1 | Std. Mean Difference (IV, Random, 95% CI) | -5.09 [-7.35, -2.83] |  |
|  | **Animal species** |  |  |  | 0% |
|  | Rat | 2 | Std. Mean Difference (IV, Random, 95% CI) | -1.08 [-1.79, -0.36] | 0% |
|  | Mice | 3 | Std. Mean Difference (IV, Random, 95% CI) | -0.61 [-3.61, 2.40] | 93% |
|  | **Cancer types** |  |  |  | 92.6% |
|  | Lung cancer | 1 | Std. Mean Difference (IV, Random, 95% CI) | -5.09 [-7.35, -2.83] |  |
|  | Gastric cancer | 2 | Std. Mean Difference (IV, Random, 95% CI) | -1.08 [-1.79, -0.36] | 0% |
|  | Sarcoma | 1 | Std. Mean Difference (IV, Random, 95% CI) | 1.76 [0.69, 2.82] |  |
|  | Lymphoma | 1 | Std. Mean Difference (IV, Random, 95% CI) | 0.93 [-0.20, 2.05] |  |
|  | **Moxibustion methods** |  |  |  | 0% |
|  | Direct moxibustion | 2 | Std. Mean Difference (IV, Random, 95% CI) | -2.00 [-7.89, 3.89] | 95% |
|  | Suspended moxibustion | 3 | Std. Mean Difference (IV, Random, 95% CI) | -0.15 [-1.99, 1.68] | 89% |
|  | Total | 5 | Std. Mean Difference (IV, Random, 95% CI) | -0.73 [-2.42, 0.96] | 90% |
